# Impact of joint contractures on functioning and social participation in older individuals – development of a standard set (JointConFunctionSet): study protocol

**DOI:** 10.1186/1471-2318-13-18

**Published:** 2013-02-21

**Authors:** Martin Müller, Uli Fischer, Gabriele Bartoszek, Eva Grill, Gabriele Meyer

**Affiliations:** 1 Institute for Medical Information Processing, Biometrics and Epidemiology, Ludwig-Maximilians-Universität München, Munich, Germany; 2 School of Nursing Science, Witten/Herdecke University, Witten, Germany

**Keywords:** Contracture (MeSH), Aged (MeSH), Aged, 80 and over (MeSH), Disabled persons (MeSH), Outcome assessment (Health care) (MeSH), Geriatric rehabilitation, Home care (MeSH), Nursing homes (MeSH), Acute hospital care

## Abstract

**Background:**

Joint contractures are frequent in older individuals in geriatric care settings. Even though they are used as indicator of quality of care, there is neither a common standard to describe functioning and disability in patients nor an established standardized assessment to describe and quantify the impact of joint contractures on patients’ functioning. Thus, the aim of our study is (1) to develop a standard set for the assessment of the impact of joint contractures on functioning and social participation in older individuals and (2) to develop and validate a standardized assessment instrument for describing and quantifying the impact of joint contractures on the individuals’ functioning.

**Methods:**

The standard set for joint contractures integrate the perspectives of all potentially relevant user groups, from the affected individuals to clinicians and researchers. The development of this set follows the methodology to develop an International Classification of Functioning Disability and Health (ICF) Core Set and involves a formal decision-making and consensus process. Evidence from four preparatory studies will be integrated including qualitative interviews with patients, a systematic review of the literature, a survey with health professionals, and a cross sectional study with patients affected by joint contractures. The assessment instrument will be developed using item-response-theory models. The instrument will be validated.

**Discussion:**

The standard set for joint contractures will provide a list of aspects of functioning and health most relevant for older individuals in geriatric care settings with joint contractures. This list will describe body functions, body structures, activities and participation and related environmental factors. This standard set will define what aspects of functioning should be assessed in individuals with joint contractures and will be the basis of the new assessment instrument to evaluate the impact of joint contractures on functioning and social participation.

## Background

Free movement of the limbs is a prerequisite of mobility and autonomy in old age. Joint contractures, i.e. restrictions in full range of motion of any joint due to deformity, disuse or pain, are common problems of frail older people, particularly in nursing home residents [1]. Contractures are among the most unexplored and underreported syndromes in clinical and homecare settings. Epidemiological studies indicate a wide range of prevalence of joint contractures in older individuals between 20% and 80% [2-4]. This variation is due to different definitions of contracture and varying diagnostic criteria or data collection methods, different research settings, sample size and study participants’ characteristics [5]. The aetiology of joint contractures is multifaceted. In older people contractures may be caused by a variety of health conditions and situations, but immobility due to an acute injury or disease seems to be the major risk factor [6].

Upper limb joint contractures may result in loss of ability to dress or eat independently while lower limb contractures may lead to instability and inability to walk independently and higher risk of bed confinement [5,7]. Joint contractures further increase the risk of other adverse patient outcomes like pain, pressure ulcers and risk of falls [8]. Thus, joint contractures are a major cause for excess disability in older people with a significant impact on overall quality of life and functioning. Preventive and rehabilitation interventions targeting joint contractures may decrease morbidity, increase functioning and quality of life, and, ultimately, prevent long-term disability.

In the United States of America, presence of joint contractures is an established indicator of quality of care in nursing facilities [1,9]. In Germany, joint contracture risk assessment and prevention have recently been defined as a quality indicator of nursing home care that should be regularly monitored by experts from the statutory health insurance system. Nursing homes are obliged to report whether they regularly assess the risk of joint contracture and administer relevant preventive measures [10,11]. In clinical settings, joint contractures are assessed by measuring the range of motion. However, from a patient- and nursing-oriented perspective the relevance of a systematic registration of contractures in care-dependent older people is unclear unless their impact on functioning is understood. Contracture assessment is only an intermediate step in the evaluation of patient-relevant outcomes such as quality of life, functioning, and the ability to participate in everyday life and social participation.

Arguably, a clinical definition of joint contracture is difficult because the contracture’s severity is determined by the consequences on activities of daily living, quality of life and social participation. In addition, there is no consensus on aspects most relevant to the affected individuals. A variety of functional measures is currently used for the assessment and evaluation of geriatric patients. To date, there is no consensus on common concepts for the choice of outcome measures specifically for evaluating the impact of interventions targeted on joint contractures. Reliable data on individuals’ (and families’) burden due to joint contracture are a prerequisite for the development of tailored interventions targeted to vulnerable groups and specific situations.

Considering that interdisciplinary collaboration is a key aspect of rehabilitation quality, and that assessment is one of the basic features of this collaboration, a common conceptual basis needs to take into account the perspectives of different health professionals involved as well as the perspective of the affected individuals. The International Classification of Functioning, Disability and Health (ICF) is likely to be a suitable common framework. Based on the ICF it is possible to select sets of categories, the ICF Core Sets [12], out of the whole classification which can serve as minimal standards for the assessment of the consequences of contractures on functioning. One established ICF Core Set, the ICF Core Set for patients in geriatric post-acute rehabilitation facilities [13] – which may cover a large fraction of aspects of functioning and disability relevant to patients with joint contracture –, will have to be taken into account for this.

The aim of our study is (1) to develop a standard set for the assessment of the impact of joint contractures on functioning and social participation in older individuals and (2) to develop and validate a standardized assessment instrument for describing and quantifying the impact of joint contractures on the individuals’ functioning.

## Methods/design

The ICF classifies domains of functioning, along with their contextual factors, which are encountered in human life [14]. As such, the ICF will be the basis of our study. To address all potentially relevant risk factors for joint contractures, we will apply the well-established methodology of developing ICF Core Sets to the health care problem of joint contractures [12]. ICF Core Sets are selections of ICF categories from the entire classification which are relevant to specific health conditions or care situations. Specifically, the ICF Core Sets are developed in a formal decision-making and consensus approach, integrating evidence from four preliminary studies:

(1) Qualitative interviews with individuals with joint contractures and their caregivers will be carried out to explore aspects of functioning and health, which are important to the affected individuals and their significant others.

(2) An expert survey will be performed via an online platform to gather the opinion of international experts from different professions regarding the most relevant and typical areas of functioning and health to be considered in individuals with joint contractures.

(3) A multicentre cross-sectional study with individuals with joint contractures will be performed to describe the prevalence of limitations and restrictions in functioning and health in individuals with joint contractures in geriatric care settings.

(4) A recently performed systematic review [15] will be updated and re-analysed to extract categories of the ICF from the outcome measures used.

**Figure 1:**
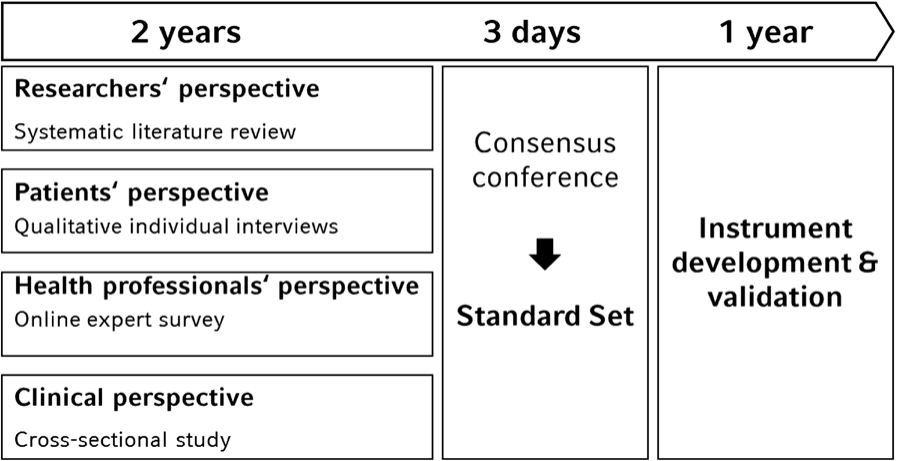
Process of developing the standard set, instrument development and validation.

The information collected in the four preparatory studies will be presented at a consensus conference [12]. Experts in the field of joint contractures including nurses, physicians, physical therapists, occupational therapists (both researchers and clinicians) and patients’ representatives will be invited to work actively together in order to arrive at a consensus on the most adequate categories of the ICF to be included in the standard set for joint contractures (see Figure 1).

Based on this standard set, a standardized assessment instrument for describing and quantifying the impact of joint contractures on the individuals’ functioning will be developed.

### Study designs and samples

As there is no consensual definition for identifying joint contractures, we operationalize their presence as follows: Joint contractures will be defined as restricted active and passive range of motion in at least one major joint (shoulder, elbow, wrist, hip, knee, and ankle). The presence has to be indicated by 1) physicians’ diagnosis or 2) physiotherapists’ or trained nurses’ assessment.

Qualitative interviews with affected individuals will be carried out separately for each study setting (home care, nursing home, geriatric acute wards, post-acute geriatric rehabilitation facilities) in order to be able to compare the findings between different situations of care. The sample size will be determined by saturation, i.e. the point at which an investigator has obtained sufficient information from the field. Experiences from our own earlier studies [16,17] indicate a sample size of 10 to 15 persons per setting. The sampling strategy follows the idea of theoretical sampling adopted from the grounded theory methodology [18]. This strategy aims to assure maximum sensitivity in order to gather a maximum variety of experiences from the participants. Inclusion criteria will be fluency in the German language, age ≥65 years, presence of joint contractures as defined above, MMSE ≥24 points [19,20] and written formal consent. Potential participants will be asked for willingness to participate by the staff of the cooperation partners. A short study summary and patient information will be provided. The expert survey via an online platform will involve health professionals from all relevant professions (nurses, physicians, physiotherapists, occupational therapists, social worker). Inclusion criteria are clinical expertise (work experience in patients with joint contractures >5 years in geriatric care settings, i.e. hospitals, rehabilitation facilities and nursing homes) or research expertise (relevant publication on joint contractures within the last 5 years). The fields of practice or research should be well-balanced between the experts and according to the study population. Sample size calculation with a power of 0.8 and a level of significance of 0.05 revealed a sample size of 204 experts to determine frequencies of relevant aspects of functioning and health with a precision of 10%. Based on previous experiences from studies [21], inclusion and participation of about 200 international experts within six months is likely to be feasible. We will contact national and international professional organizations to nominate experts in the field of joint contractures. At the same time, first and senior authors of the papers identified in the systematic review will be contacted and asked to participate. In addition, all participating experts will be asked to nominate further experts. The recruitment procedure has been well proven in former own studies [21,22].

For the cross-sectional survey, inclusion criteria will be age ≥65 years, presence of joint contracture as defined above and written informed consent. In order to get a representative sample of individuals with contractures data will be collected consecutively in three different settings and in two different German regions (Munich, Bavaria, and Witten, North Rhine-Westphalia). The involved facilities have large catchment areas predisposing a representative case-mix. Each of the coordinating sites will be responsible for 50% of the sample, i.e. for 100 participants. Eligible patients will be identified by the weekly team conferences held at the respective hospitals. The study nurses will then be informed. Additionally, they will participate in ward rounds of the respective hospitals and sites. Under the assumption of an equal effects model [23], a power of 0.8 and a level of significance of 0.05, a sample size of 194 individuals would be necessary in order to determine frequencies with a precision of 10%. For the instrument validation, participants will also be asked to consent to a follow-up. An overview of methods and designs is given in Table 1.

**Table 1:** Overview of the study parts

	**Aim**	**Design**	**Participants**	**Estimated sample size**
**Researchers’ perspective**	Identification of outcome measures used in studies focusing on contractures using the ICF as a reference framework	Systematic Review	Not applicable	
**Patients’ perspective**	Identification of aspects of functioning and disability relevant to older individuals with contractures using the ICF as a reference framework	Qualitative individual interviews	Individuals in nursing homes, specialized geriatric rehabilitation facilities, geriatric wards in acute hospitals	Approx. 30 depending on data saturation
**Health professionals’ perspective**	Identification of aspects of functioning and disability relevant in the management and treatment of individuals with contractures from the perspective of experienced professionals using the ICF as a reference framework	Online expert survey	Health professionals with >5 years experience (nurses, physicians, physiotherapists, occupational therapists)	Approx. 200
**Clinical (epidemiological) perspective**	To describe functioning and disability of individuals with vertigo and to identify the most common problems using the ICF	Cross-sectional study	Individuals in nursing homes, specialized geriatric rehabilitation facilities, geriatric wards in acute hospitals	Approx. 200
**Instrument validation**	To examine inter-rater-reliability and predictive validity of the developed instrument	Cross-sectional study	Individuals in nursing homes, specialized geriatric rehabilitation facilities, geriatric wards in acute hospitals	Approx. 30

Approval of the study was obtained from the responsible ethics committees prior to start (Ethics committee of the medical faculty of the Ludwig-Maximilian-Universität in Munich, 530–12 and 087–12, and Ethics committee of the German Society of Nursing Science, January 25, 2013).

### Data collection

Data for the qualitative study will be gathered by face-to-face interviews based on an established interview guideline [17,24]. The interviews will be audio-recorded and transcribed verbatim.

Data collection for the expert survey will be conducted via an online platform based on the Delphi Method [25]. The experts will be asked to list the impairments, limitations and restrictions in body functions and structures and activities and participation, as well as relevant environmental and personal factors of individuals with joint contractures.

In the cross-sectional study, data on functioning and health will be collected using the ICF check list [26] in structured interviews. In cases of individuals with communication impairments, data will be collected in a proxy interview with the affected relatives or nurses in charge. In addition, individuals and health professionals in charge are requested to evaluate health and functioning using a rating scale ranging from 10 (excellent/no problems) to 0 (poor/complete problems). This will serve as an outcome for multiple analyses. Socio-demographic and condition-specific data, such as marital status, main medical diagnosis or duration of in hospital stay will also be recorded. In order to gain a comprehensive view on the consequences, data will be collected from individuals who are about to be discharged from acute geriatric wards, from individuals in post-acute rehabilitation facilities, from individuals in nursing homes and in home care situations. Items of the outcome measures used in the study retrieved by the recent systematic review by Gnass et al. (2010) will be identified using a standardized procedure [27].

The consensus conference follows an established procedure of formal decision making and consensus building integrating the results from the previous studies [12,28]. According to extensive former experiences, it will involve about 30 expert experts in the field of joint contractures including clinicians and researchers from all relevant professions as well as consumer representatives.

The developed standardized assessment instrument will be tested at the study sites participating in the cross-sectional study. Participants will be asked to fill in the instrument and will be contacted again after discharge for a follow up assessment.

### Analysis

Qualitative content analysis following a descriptive approach will be used to analyse the content of the qualitative interview [29]. The retrieved aspects of functioning and health will be translated into categories of the ICF [27]. The result of this analysis will be a list of ICF categories relevant for affected individuals and their caregivers.

The results of the expert survey will be translated into categories of the ICF using a standardized procedure [27]. Frequencies of ICF categories and their 95% confidence intervals will be calculated.

All items of the outcome measures extracted from the systematic review will be translated into categories of the ICF following a standardized approach [27]. Frequencies of ICF categories and their 95% confidence intervals will be calculated.

For cross-sectional analyses, absolute and relative frequencies (prevalence) of impairment, limitation or restriction alongside 95% confidence intervals will be calculated. To identify potential confounders, analyses will be stratified for age groups and sex. Based on previous experiences in ICF Core Set development and validation studies, only few subjects are expected to have missing values.

To develop an instrument for describing and quantifying the impact of joint contractures on the individuals’ functioning, we will apply Item Response Theory-modelling to the data of the cross-sectional-study and the knowledge from the consented standard set. By using Item Response Theory methods [30] one can examine whether the selected categories cover a common underlying trait (such as disability following joint contractures), thus forming a scale, or which of the selected categories have a reasonable fit in relation to the assumed trait, or whether the selected categories cover the spectrum of ability one is likely to encounter in typical affected individuals. The approach has been shown to result in valid scales [31]. We will test the inter-rater reliability of the resulting instrument in a validation sample with repeated measurements. The optimal sample size for reliability testing will be determined by feasibility and precision considerations. The experiences gathered from other reliability studies involving ICF Core Sets [32] have shown that this can be done, even given a very high or very low proportion of positive ratings, with a sample size of n = 30 to detect a moderate kappa (0.5–0.6) with a power of 0.8. Internal consistency will be examined by Cronbach’s alpha. Since there is no criterion measure available, criterion validity will be examined by means of predictive validity of the instrument. This predictive utility will be examined by investigating whether the new instrument is able to predict future participation restriction. Eligible participants from the sites of the cross-sectional study will be followed up after discharge by phone calls or home visits to examine participation restriction. We will use the Impact on Autonomy and Participation (IPA) questionnaire for validation. This is a generic questionnaire focusing on self-perceived restriction in participation associated with health condition or disability [33]. The IPA covers the main aspects of the component Activities and Participation as described by the ICF [34]. A German version of the IPA has recently been validated. The IPA consists of eight subscales with a total of 41 items: self-care and appearance, mobility, leisure, social relationships, work, education, family role, financial independence. Each item is scored on a five-point rating scale, ranging from 1 (excellent) to 5 (very poor). For each subscale, a standardised summary measure can be calculated based on the item scores weighed by the number of items, where a higher score indicates a greater perceived handicap. The score developed from the cross-sectional data will be investigated as to whether it is able to predict participation restriction in this sample of older persons. The predictive validity will be analysed by regression models where perceived participation serves as a dependent variable and the ICF measure as an independent variable.

## Discussion

The standard set for joint contractures will provide a list of aspects of functioning and health most relevant for older individuals with joint contractures. This list will contain body functions, body structures, activities and participation and related environmental factors. This set will define what aspects of functioning should be assessed in individuals with joint contractures and will be the basis of a new instrument. This instrument should assess the consequences of joint contractures and will provide a clinical and scientific basis to study and therefore understand the impact of this condition in older individuals in geriatric care settings. In the clinical situation, the instrument will lead to better care of patients since it allows to assess the impact of the condition from a patient-centred perspective and therefore to choose the adequate treatment. It will also allow evaluating treatment strategies and comparing them amongst each other in a meaningful way, or might be the basis for developing new treatment programmes based on the knowledge about aspects really relevant to the affected individuals.

## Competing interests

The authors declare that they are no competing interests.

## Authors’ contributions

MM, EG, GM and GB contributed to the conception of the study and applied for funding. MM and EG conceived the study design, GM and GB contributed to the study design. MM and EG drafted the manuscript. UF, GM and GB critically revised the drafts and contributed to the final writing of the paper. All authors read and approved the final manuscript.

## Pre-publication history

The pre-publication history for this paper can be accessed here:

http://www.biomedcentral.com/1471-2318/13/18/prepub
